# Molecular Modelling of Islet β-Cell Adaptation to Inflammation in Pregnancy and Gestational Diabetes Mellitus

**DOI:** 10.3390/ijms20246171

**Published:** 2019-12-06

**Authors:** Petra I. Lorenzo, Alejandro Martín-Montalvo, Nadia Cobo Vuilleumier, Benoit R. Gauthier

**Affiliations:** 1 Andalusian Center for Molecular Biology and Regenerative Medicine, CABIMER (Junta de Andalucía-CSIC-Universidad de Sevilla-Universidad Pablo de Olavide), Calle Américo Vespucio, 24, 41092 Sevilla, Spain; alejandro.martinmontalvo@cabimer.es (A.M.-M.); nadia.cobo@cabimer.es (N.C.V.); 2 Biomedical Research Network on Diabetes and Related Metabolic Diseases-CIBERDEM, Instituto de Salud Carlos III, 28029 Madrid, Spain

**Keywords:** pregnancy, inflammation, fetal growth alteration, gestational diabetes, gestational hypertension, PAX4, PAX8, HMG20A

## Abstract

Gestational diabetes mellitus (GDM), a metabolic disease that develops with the increase in insulin resistance during late pregnancy, is currently one of the most common complications affecting pregnancy. The polygenic nature of GDM, together with the interplay between different genetic variants with nutritional and environmental factors has hindered the full understanding of the etiology of this disease. However, an important genetic overlap has been found with type 2 diabetes mellitus (T2DM) and, as in the case of T2DM, most of the identified loci are associated with β-cell function. Early detection of GDM and adequate interventions to control the maternal glycemia are necessary to avoid the adverse outcomes for both the mother and the offspring. The in utero exposure to the diabetic milieu predispose these children for future diseases, among them T2DM, originating a vicious circle implicated in the increased prevalence of both GDM and T2DM. The involvement of inflammatory processes in the development of GDM highlights the importance of pancreatic β-cell factors able to favor the adaptation processes required during gestation, concomitantly with the protection of the islets from an inflammatory milieu. In this regard, two members of the Pax family of transcription factors, PAX4 and PAX8, together with the chromatin remodeler factor HMG20A, have gained great relevance due to their involvement in β-cell mass adaptation together with their anti-inflammatory properties. Mutations in these factors have been associated with GDM, highlighting these as novel candidates for genetic screening analysis in the identification of women at risk of developing GDM.

## 1. Metabolic Alterations During Pregnancy: Gestational Diabetes Mellitus

Pregnancy is a period of metabolic plasticity with transient mild insulin resistance as a physiological adaptation to ensure the preferential use of the circulating glucose by the fetus, to prioritize, in this way, fetal growth [1]. During normal pregnancies, maternal sensitivity to insulin is decreased by approximately 50%, which is counteracted by an increase of approximately 250% in maternal insulin production to maintain the euglycemia [2,3]. This compensatory mechanism is mediated by the stimulation of β-cell mass expansion as well as an increase in insulin production and secretion by β-cells [4]. When these adaptive processes are unable to compensate for the increase in insulin demand, maternal blood glucose levels rise, resulting in the development of gestational diabetes mellitus (GDM). Thus, based on the American Diabetes Association (ADA) criteria, GDM is defined as a subgroup of diabetes diagnosed in the second or third trimester of pregnancy that was not overt diabetes before gestation [5]. Nonetheless, these criteria may vary worldwide, for example, with the more recent rigorous recommendations elaborated by the International Association of the Diabetes and Pregnancy Study Group [6]. To avoid complications derived from the development of GDM, pregnant women are routinely tested for GDM at 24–28 weeks of gestation. Noteworthily, the important increase in the prevalence of Type 2 diabetes (T2DM) in the last decades, together with its earlier onset [7,8] has raised the number of women of childbearing age with undiagnosed T2DM or prediabetes that can develop to frank diabetes in response to the increased insulin demand that takes place during pregnancy. In these cases, diabetes can develop as early as in the first trimester of pregnancy and is not considered GDM but undiagnosed preexisting pregestational diabetes [5]. Therefore, pregnant women with risk factors for T2DM, such as a familiar history of T2DM and/or overweight/obesity should be checked in their first prenatal visit [5]. The early detection of diabetes during pregnancy will allow an earlier intervention (changes in lifestyle and/or pharmacological treatment) that will ensure the normoglycemic status of the mother during the pregnancy, avoiding in this way the in utero exposure of the fetus to a hyperglycemic detrimental environment.

Currently GDM is one of the most common complications during pregnancy, with a prevalence reaching up to 27.5% in Southern Italian and an alarmingly 41.9% in Northern Indian women using the new and tighter diagnostic criteria recently adopted by the International Association of the Diabetes and Pregnancy Study Group [6,9,10,11]. The growing prevalence of GDM is due, in part, to the increase in maternal overweight and the later age of conception [12,13,14]. Nevertheless, although obesity increases GDM incidence 1.3–3.8 times [15], 20–30% of the women who develop GDM are not obese, suggesting that other factors, including physiologic factors and age-related insulin resistance, play a role in the development of this disease [10]. In this regard, women with subclinical hypothyroidism are at a significantly greater risk of developing GDM [16] while decreased levels of free thyroxin (FT4) during early pregnancy have been correlated with an increased incidence of GDM [17,18]. Interestingly, T4 supplementation has been shown to increase circulating insulin levels as well as glucose clearance in mice and to delay hyperglycemia in a mouse model of autoimmune diabetes, correlating with an increase in β-cell proliferation [19].

## 2. Etiology of GDM and Genetic Component of the Disease

GDM is a polygenic disease, precipitated by the interplay between different genetic variants in combination with nutritional and environmental factors. As such, the underlying etiology and mechanisms have remained elusive, hindering the development of accurate diagnostic tools. Given the genetic component of this disease, the identification of polymorphisms/mutations that correlate with a higher risk of GDM has received significant attention. The similar pathology of GDM and T2DM, characterized by impaired insulin secretion and increased insulin resistance, together with the strong relationship between these two types of diabetes, has prompted candidate gene approach studies, prioritizing T2DM-susceptibility variants, for the identification of polymorphisms associated with an increased risk of GDM development [20]. Different case-control studies and meta-analysis using single nucleotide polymorphisms (SNPs) associated with T2DM indicate a significant overlap in the genetic architecture between GDM and T2DM, substantiating the premise that both clinical manifestations share common pathogenic pathways [21,22,23,24]. Moreover, a recent study has shown that the inclusion of 8 common T2DM SNPs in the prediction of GDM increases the sensitivity and specificity of the prediction based solely on clinical factors [25]. The fact that a Genome Wide Association Study (GWAS) performed in a South Korean cohort reported a strong association with GDM of several SNPs in different genes that were already known to be T2DM susceptibility genes [26] supports the strong genetic overlap between both forms of diabetes. Interestingly, the majority of loci associated with GDM impact β-cell function [27]. Nevertheless, the association with GDM was not demonstrated by all T2DM associated loci [28], suggesting that the effect size of the genetic variants could be different between the two conditions and that some of the identified loci could be specific to each disease. In support of this premise, several GWAS studies have identified variants in the *HKDC1* and *BACE2* genes influencing glycemic traits during pregnancy as well as being specifically associated with GDM in several ethnic groups [29,30,31]. Further studies are needed to reveal the degree of genetic overlap between GDM and T2DM to fully elucidate the interaction between these two forms of diabetes.

Additionally, Maturity Onset Diabetes of the Young (MODY) a monogenic form of diabetes that accounts for 5% of all diabetes cases, can also be misdiagnosed as GDM [20]. For example, individuals with mutations in the glucokinase gene (MODY2) may remain asymptomatic until the development of insulin resistance during pregnancy and thus misdiagnosed as GDM [32]. Currently 15 different MODY genes have been identified (HNF4A; GCK; HNF1A; PDX1; HNF1B; NEUROD1; KLF11; CEL; PAX4; INS; BLK; ABCC8; KCNJ11; APPL1; RFX6), presenting age-dependent penetrance pending location of mutations [33]. A proper diagnosis of these types of monogenic diabetes is critical for accurate etiology-based treatment, which may lead to better glucose control and reducing both pregnancy complications as well as the effects in the offspring later in life.

## 3. Adverse Outcomes of GDM

GDM is well known to cause complications during pregnancy, both to the mother, such as hypertensive disorders of pregnancy (including pre-eclampsia and gestational hypertension) and often resulting in cesarean delivery, as well as to the infant, such as large-for-gestational-age infants, macrosomia, preterm birth, respiratory distress syndrome, neonatal hypoglycemia and neonatal jaundice [1,10,34]. In addition, GDM significantly increases the maternal postpartum risks of metabolic syndrome, T2DM, insulin resistance, hypertension, cardiovascular disease, renal damage and serious liver disease [35,36,37,38,39,40]. Noteworthily is the strong increase in the prevalence of T2DM among women with a history of GDM, among who up to 70% can develop T2DM later in life [41,42]. Therefore, women who developed GDM should be monitored for T2DM, including testing for persistent diabetes at 4–12 weeks postpartum as well as lifelong screening for prediabetes and T2DM [5]. The early detection of prediabetes/T2DM will allow earlier interventions that will reduce the complications associated with this disease.

GDM is also associated with adverse outcomes for the progeny, such as respiratory distress syndrome, metabolic and cardiac dysfunction, obesity, brain disorders [39,43,44,45]. In general, a higher prevalence of obesity and diabetes, more than what genetics alone would predict, was observed in the children from women with diabetes during pregnancy, when compared to children from nondiabetic women [10,46,47]. This fact, together with the observation of a greater transmission of T2DM from the mother than from the father [48], revealed the important role of in utero exposure to a diabetic environment in the offspring outcome. Supporting this, studies in Pima Indians, a population with a high prevalence of T2DM, have revealed sibling discrepancies, with a higher incidence of diabetes among siblings born after maternal diagnosis of diabetes when compared to siblings born before the maternal diagnosis of diabetes [48]. Nevertheless, caution should be taken, as women with diabetes during pregnancy are more likely to be overweight or obese, and obesity *per se* has adverse effects on the offspring [49]. In order to determine the specific role of the hyperglycemia *per se* in the adverse outcomes of the progeny, the HAPO (Hyperglycemia and Adverse Pregnancy Outcome) study, analyzing a multiethnic cohort of 25,505 pregnant women with glucose levels below GDM threshold at 15 centers in 9 countries, demonstrated that maternal glucose levels during pregnancy were associated with birth weight and newborn adiposity [50]. A recent follow up of this study with children with a mean age of 11.4 years has substantiated the link between maternal glucose levels and childhood adiposity [51]. Interestingly, childhood adiposity increases the susceptibility of obesity in adolescence and adulthood, and therefore the subsequent adverse metabolic outcomes [52,53]. Thus, the development of diabetes during gestation results in a higher susceptibility to obesity and diabetes in the new generation, which in turn will increase the susceptibility of GDM, resulting in a vicious circle. These data support the theory of the Developmental Origins of Health and Disease (DOHaD), which states that environmental factors during fetal life have important effects on the susceptibility to disease development later in life [54].

Epigenetic modifications such as DNA methylation, histone modification and noncoding RNAs have become a likely link between the in utero exposure to a diabetic environment and the adverse outcomes for the offspring. Indeed, there is an association of GDM and diabetes during pregnancy with the epigenetic status of the exposed offspring [39,55,56]. Importantly, these epigenetic modifications, despite not altering the genome of the organism, can be mitotically stable over time, producing long-term changes in gene expression [57].

## 4. Inflammation Underlying GDM Development and Derived Adverse Outcomes

Pregnancy induces a dynamic and highly regulated inflammatory profile necessary for proper implantation and allows fetal development [3,58]. However, in pregnant women who develop GDM, evidence of inflammatory dysregulation can be detected early during pregnancy [3]. Inflammation, a process initially triggered to restore tissue homeostasis after an injury, may become chronic and pathological when it is not properly resolved. In this regard, obesity and metabolic diseases are associated with a chronic, low-grade inflammation, termed meta-inflammation, that alters the immune profile favoring a pro-inflammatory environment in several tissues such as adipose, liver, kidney, heart and pancreas [59,60]. GDM has been correlated with an increase in circulating pro-inflammatory cytokines (IL-1β, IL-6, TNFα and leptin) and a decrease in anti-inflammatory molecules (IL-4, IL-10 and adiponectin) pinpointing to an important role of inflammation in the pathophysiology of GDM [3,61,62]. Analysis of the peripheral T-cell profile in the third trimester of GDM pregnancies revealed a higher proportion of Th2, Th17 and Treg cells that persisted up to six months post-delivery [63].

The low-grade inflammation that characterizes obesity and T2DM/GDM with a mild increase in circulating pro-inflammatory cytokines can impact β-cell function/survival as shown in in vitro studies. Cytokine treatment of islets isolated from prediabetic mice, at doses comparable to that observed in T2DM, decreased their capacity to secrete insulin in response to high glucose conditions [64]. Additionally, in response to free fatty acids (FFAs) β-cells initiate macrophage recruitment through the production of chemokines [65]. In agreement with this, immunohistochemical analyses of pancreatic+s from T2DM patients have demonstrated a significant increase in immune cell infiltration of the islets [66], mainly composed of macrophages, likely of the pro-inflammatory M1 subtype [60]. The resident macrophages of islets, that under normal conditions can exert homeostatic and regenerative properties can become pro-inflammatory under pathologic conditions such as obesity and/or diabetes. Noteworthily, the in vivo maintenance of macrophages anti-inflammatory phenotype could be achieved by blocking IL-6 signaling or by treatment with IL-4 and IL-10 [67]. However, IL-6 is increased in GDM while IL-4 and IL-10 are in general decreased, thus suggesting that the GDM environment favors macrophages transition to a pro-inflammatory M1 phenotype. *In vitro* analysis of the mechanism underlying the switch of macrophages from anti-inflammatory M2 to pro-inflammatory M1 phenotype have shown that under high glucose conditions the phagocytosis of apoptotic β-cells causes macrophages switch from M2 to M1 phenotype, correlating with the activation of the NLRP3-inflammasome that triggers IL-1β release and increases ROS production [68]. In agreement with a role for NLRP3-inflammasome and subsequent increase in IL-1β during GDM, the inhibition of the pancreatic NLRP3 inflammasome in a GDM mouse model resulted in improved glucose homeostasis [69]. Moreover, in vitro treatment of human and mouse islets with IL-1β, a cytokine that is increased in GDM [61], causes β-cell dedifferentiation and dampened insulin secretion capacity [66]. Additionally, the chronic exposure of isolated human islets to leptin, an adipokine also increased during GDM, blunted on one hand the production of the IL-1 receptor antagonist (IL-1Ra), that inhibits IL-1β signaling, and on the other hand induced IL-1β release leading to impaired β-cell function, caspase-3 activation and apoptosis [70]. Moreover, low levels of the antidiabetic adipokine adiponectin have been associated with β-cell dysfunction in women with GDM [71]. Studies in mice have recently shown that this adipokine is involved in β-cell expansion during gestation, without affecting the secretory function of the β-cells [72]. Altogether these data establish a crucial role of inflammation in the pathogenesis of GDM.

It has been suggested that inflammation in pregnant obese women or with GDM may influence fetal development [73]. Several lines of evidence from experimental animal models as well as from clinical studies indicate that maternal and placental inflammation associated with GDM and obesity can affect neurodevelopment and cause alterations in the inflammatory responses in their offspring [74,75]. The induction of GDM in a mouse model exacerbates the response of animals to experimentally induced maternal immune activation (MIA), with important consequences in brain development in the offspring. Additional studies in mice have shown that IL-6, which is increased in GDM correlating with fasting and postprandial circulating glucose [62], is crucial in MIA-dependent behavioral alterations in the offspring [76,77]. Moreover, hyperglycemia and hyperinsulinemia, situations produced during diabetes, can increase systemic inflammation and exaggerate and/or prolong responsiveness to pro-inflammatory stimuli, supporting a possible interaction of GDM and MIA. These data suggest that children born to mothers with GDM, exposed to midgestation infections/immune activation, have increased vulnerability for developmental disorders [78].

**Figure 1:**
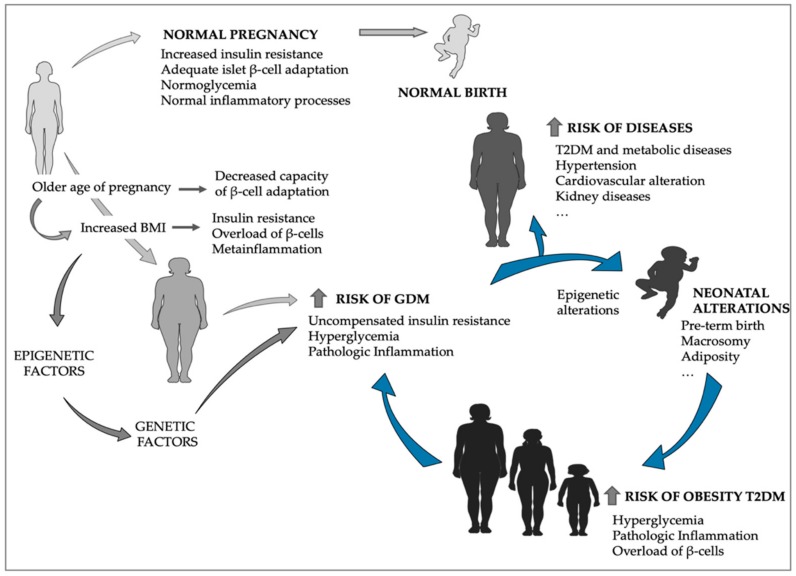
Gestational diabetes mellitus (GDM) derived effects in offspring as precursors of increased type 2 diabetes mellitus (T2DM) and GDM prevalence. The older age of pregnancy and the increase in maternal BMI are important risk factors for GDM development, which results in increased incidence of diseases for the mother later on in life, as well as for the offspring, who are primed for obesity and T2DM, that in turn will increase next-generation prevalence of GDM and thus T2DM. (The figure was generated using Servier Medical Art templates: Available online: http://www.servier.com).

All together these data reveal the imperative need of finding better treatments for GDM as well as defining novel and more effective early markers of GDM to avoid not only the subsequent maternal complications of GDM, but also to reduce the striking effects on the offspring derived from the in utero exposure to the diabetic milieu (Figure 1). Adequate early detection of a prediabetic status in pregnant women will allow intervention studies that will impede the development of hyperglycemia and therefore prevent future development of T2DM and other metabolic diseases in the children born from these pregnancies, limiting in this way the vicious cycle of diabetes across generations.

## 5. Candidate Factors for GDM Prevention Should Convey β-Cell Adaptation and Attenuation of Inflammation

GDM can be broadly defined as an inadequate environmental/physiological adaptation of islets, either functionally or in mass, to compensate for the increase in insulin demand that takes place during pregnancy. As aforementioned, GDM is often associated with the increase in maternal BMI as well as with late age pregnancies. In the first case, the insulin resistance status provoked by the overweight/obesity of the mother will be further enhanced by the pregnancy, overloading the β-cells and leading to a more rapid deterioration of β-cell functionality and plasticity. In the second case, the reported loss of β-cell expansion/plasticity with age likely accounts for a reduced adaptive response of the islets and subsequent increased susceptibility to develop GDM. In both situations, pregnancy-induced immune alterations will compound to precipitate the development of GDM. However, similar to T2DM, not all overweight/obese and/or older women develop GDM, indicating that an individual’s genetic makeup will influence the physiological or pathophysiological outcome. Although several SNPs have been associated with GDM, functional data validating the functional impact of these SNPs have yet to emerge in the context of pregnancies and inflammation [23,24]. In contrast, using a candidate gene approach, we have identified two members of the paired box (Pax) family of transcription factors, PAX4 and PAX8 as well as a high mobility group member HMG20A as important regulators of islet function and adaptation to stress conditions that include GDM. Herein we provide an overview of the mode of action of these factors in islet physiology and discuss/argue the potential role of genetic/mutant variants in the maladaptation of the β-cell mass in response to pregnancy and the inflammatory response associated with this state that may lead to GDM.

### 5.1. PAX4, a Master Regulator of β-Cell Phenotype and Plasticity That Is Upregulated During Pregnancy

PAX4 is a master regulator of β-cell commitment during development as well as for β-cell plasticity during adulthood [79,80,81]. In mice, the lack of this transcription factor during development causes neonatal death accompanied by severe hyperglycemia. Immunohistochemical analysis of the pancreas of these mice revealed the absence of islet β- and δ-cells, together with an increase in α-cell mass [82,83]. In contrast, ectopic expression of *Pax4* in the early pancreatic epithelium, or endocrine committed precursor cells induces the formation of β-cells at the expense of all other islet cell phenotypes [84]. These data highlight the crucial role of PAX4 in β-cell formation during development. Additionally, ectopic expression of *Pax4* in murine adult islet β-cells, both in vivo and in vitro, confers protection against apoptosis induced by cytokines, streptozotocin (STZ) and endoplasmic reticulum (ER)-stress [85,86], suggesting that PAX4 plays a crucial role orchestrating the regulation of β-cell molecular networks to ensure cell survival and adaptation to environmental changes. Moreover, increased expression of *Pax4* in murine islet β-cells stimulates the proliferative capacity of the β-cells favoring the expansion of the β-cell mass when it is needed [86,87]. In agreement with this, in vivo, β-cell destruction by STZ treatment of mice upregulates endogenous *Pax4* expression [88], likely to enhance the expansion capacity of the remaining β-cell. Therefore, altered expression/functionality of this transcription factor will render the organism more susceptible to environmental diabetogenic stressors, conveying not only a defective adaptation of the β-cells but also a reduced initial β-cell mass. In agreement with this, human population studies have revealed that polymorphisms/mutations in the *Pax4* gene are associated with the development of both T1DM and T2DM and consequently this transcription factor has recently been classified as a MODY gene, MODY9 [89].

**Figure 2:**
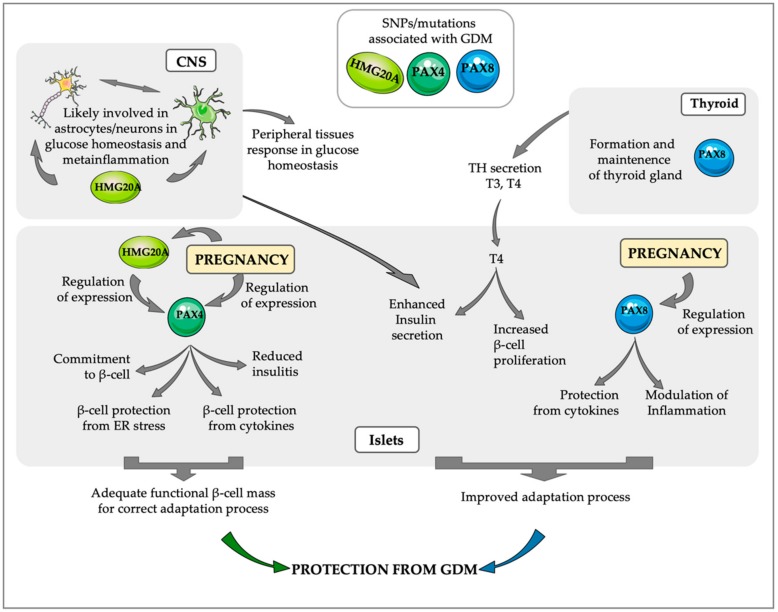
PAX4, HMG20Aand PAX8 regulated pathways that may be implicated in the protection against GDM. A transient increase in PAX4 expression during pregnancy, correlating with the increase in β-cell proliferation, confers higher protection to β-cells against ER stress and inflammation, ensuring the maintenance of an adequate functional β-cell mass able to counteract the increase in insulin resistance that takes place during pregnancy. HMG20A, one of the regulators of PAX4 in islets is also modulated during pregnancy, correlating with functional maturation of β-cells. Interestingly, HMG20A has important roles in the Central Nervous system (CNS), likely regulating the interaction between astrocytes and neurons during central regulation of glucose homeostasis. During pregnancy PAX8 expression in islets increases their protection from cytokines and is likely involved in the modulation of the inflammatory response. Additionally, PAX8 is a key factor for thyroid gland maintenance and secretion of thyroid hormones (THs), which increase β-cell proliferation and enhance insulin secretion. The important role of these three factors PAX4, HMG20A and PAX8 during pregnancy is supported by the association of SNPs/mutations in these factors with the development of GDM.

Direct in vivo modulation of PAX4 expression/functionality has been challenging, hindering advances in the therapeutic field. As such, we performed a transcriptome analysis after in vivo overexpression of PAX4 in murine β-cells aiming to open new venues of PAX4 pathways regulation by identifying novel “druggable” downstream targets of PAX4 [81,90]. Interestingly, among the identified downstream targets of PAX4 we have uncovered several immune modulators such as galectin-9 (*Lgals9*) and *Reg3g*, both of which are able to prevent/delay the apparition of hyperglycemia in the non-obese diabetic (NOD) mouse model of autoimmune diabetes correlating with decreased insulitis [91,92]. Moreover, ectopic expression of *Lgals9* in transplanted islets reduced the insulitis and prolonged grafts survival [93]. In parallel, increased *Reg3g* expression was shown to stimulate islet cell proliferation [92]. In a previous in vivo study, we also observed that overexpression of *Pax4* in β-cells resulted in higher expression levels of *Il-1Ra* expression with a concomitant increase in *Il-1β* expression therefore contributing to the maintenance of an anti-inflammatory ratio in IL-1β/IL-1Ra [85]. Besides, previous in vitro studies have shown that PAX4 acts as a repressor of human islet amyloid polypeptide (hIAPP) through its binding to a region located in proximity to the transcriptional start site (TSS) of hIAPP [94]. Noteworthily, hIAPP that is co-secreted with insulin can form amyloid deposits within the islet that trigger inflammation through the activation of the NLRP3 inflammasome in infiltrated macrophages and generation of IL-1β [95]. Altogether, these data reveal important anti-inflammatory properties of PAX4. In agreement with this, in vivo overexpression of *Pax4* in a mouse model of autoimmune diabetes reduces insulitis protecting islets against apoptosis [90]. Therefore, we can conclude that in adult islets PAX4 is not only involved in β-cell plasticity and adaptation in response to environmental stressors but also in maintaining a local anti-inflammatory environment permissive for survival and functionality of the β-cells (Figure 2).

Studies in mouse models have revealed that endogenous PAX4 expression is not homogeneous in all islet β-cells but is only expressed in approximately 30% of β-cells. Under normal physiological conditions, the expression of this factor decreases with age correlating with the decreased proliferative capacity of islet β-cells [86]. Notwithstanding, during pregnancy the percentage of β-cells expressing PAX4 transiently increases correlating with a higher proliferation of β-cells, highlighting the role of this transcription factor in the adaptation processes that take place during gestation to compensate for the increased insulin resistance [86]. In agreement with this, recent studies have associated SNPs near or within the *PAX4* gene with GDM [96,97]. Based on the importance of PAX4 on β-cell formation and plasticity, we can presume that women harboring detrimental mutations in this transcription factor will likely have a reduced β-cell mass with a blunted adaptive response to the increase in insulin resistance, rendering these women more susceptible to develop GDM. Moreover, the immunomodulatory capacity of PAX4 will favor a more permissive environment for the maintenance of islet functionality under a situation of meta-inflammation associated with GDM. We, therefore, propose that the identification of *PAX4* gene mutations will be a good marker for the identification of women at risk of GDM, that will allow early interventions ensuring not only a better outcome for the mother but also for the next generation.

Notwithstanding, despite the beneficial effects of PAX4, long term in vivo overexpression of *Pax4* in β-cells can cause dedifferentiation of the β-cells leading to the development of hyperglycemia [85]. As such, PAX4 expression/activity must be tightly regulated to permit β-cell expansion without impairing mature function [80]. In this regard, the chromatin factor HMG20A, also known as iBRAF, has become a key element in modulating *Pax4* gene expression.

### 5.2. HMG20A, an Epigenetic Modulator Bridging CNS and Islets Response to Hyperglycemia, Is Upregulated in β-Cells During Pregnancy

HMG20A, a member of the high mobility group (HMG) box-containing genes, is an important factor in the activation of neuronal-specific genes during differentiation, through modification of the histone methylation/acetylation pattern at the promoter of neuronal genes [98]. Interestingly, another HMG box-containing gene, HMG20B has an opposite function mediating the repression of neuronal genes in non-neuronal cells and neuronal progenitors through the interaction with the LSD1-CoREST chromatin-complex that contributes to the generation of repressive chromatin by the demethylation of H3K4me1/2 and H3K9me1/2 [99,100]. Mechanistic studies have revealed that HMG20A can form heterodimers with HMG20B displacing HMG20B from the LSD1-CoREST complex and releasing the complex from the chromatin, allowing gene transcription [101]. Moreover, HMG20A can also bind to the LSD1-CoREST complex [102]. Noteworthily, one of the transcription factors that mediate the interaction of the LSD1-CoREST complex to specific DNA sites is the transcriptional repressor RE-1 silencing transcription factor (REST), also termed Neuron-Restrictive Silencer Factor (NRSF), which is ubiquitously expressed in most cells of the body with the exception of mature neurons and pancreatic β-cells [103]. Remarkably, REST is expressed in progenitors of both neurons and β-cells during development but is downregulated as neurons and pancreatic cells differentiate. Aberrant induction of REST expression in neurons or progenitors plays a role in neurodegenerative and neurodevelopmental diseases while reactivation of REST in adult mice β-cells leads to their dedifferentiation and hyperglycemia [103,104]. The LSD1-CoREST complex can also be recruited at different target genes by additional transcription factors, including SNAI1, SMAD4 GFI1/1B, INSM1 [102,105,106,107], thus HMG20A can also modulate the activity of the repressor complex at these sites.

In agreement with a possible role for HMG20A in the modulation of LSD1-CoREST activity during differentiation of islet β-cells, resembling neuronal differentiation, our recent studies have shown that HMG20A is expressed in both human and mouse islet β-cells [108]. Remarkably, exposure of isolated human and mouse islets, as well as the INS-1E insulinoma cell line, to high glucose concentration causes a transient increase in *Hmg20a* expression [108]. The stimulation of β-cell mass expansion in response to high glucose requires the maturation of these new β-cells and HMG20A will likely play an important role during this process. In agreement with this, the silencing of *Hmg20a* in the rat INS-1E cells as well as in isolated mouse islets impairs glucose-stimulated insulin secretion. This correlated with decreased expression of β-cell maturity markers such as *Ins*, *MafA* or *NeuroD* and increased expression of the β-cell plasticity gene *Pax4*, suggesting dedifferentiation of β-cells [108]. Of note, PAX4 *per se* can also inhibit the expression of *Ins* and *MafA* [80], likely further inhibiting β-cell maturation. Using immunoprecipitation assays, we have demonstrated a direct interaction of HMG20A with the promoter region of the *Pax4* gene as well as to the first exon of the gene, suggesting a direct regulation of *Pax4* expression by HMG20A [108]. Further support for the important involvement of HMG20A in the islet adaptation process arises from the fact that islets from T2DM donors, in which the adaptation processes have failed, have significantly lower expression of *HMG20A* when compared with islets from normoglycemic individuals [108]. Moreover, polymorphisms/mutations in the *HMG20A* gene have been linked to both obesity and T2DM, thus being considered a diabesity gene [109,110,111,112]. In agreement with the role of HMG20A in the adaptation process of β-cells to compensate for an increase in insulin demand, we have observed a transient upregulation of *Hmg20a* in murine pancreatic β-cells during gestation, with maximal protein expression at gestational day 14.5 [108], correlating with the decrease in PAX4 expression after the initial increase triggered by the gestation [86]. These findings are consistent with the premise that after proliferation, β-cells need to downregulate PAX4 to acquire a fully metabolic mature phenotype [80]. Supporting this likely role of HMG20A in islet adaptation processes during pregnancy, polymorphisms/mutations in *HMG20A* have also been associated with GDM as well as with postpartum abnormal glucose tolerance [11,28,113,114]. Given HMG20A function as an epigenetic modulator through regulation of the LSD1-CoREST complex, it will be of interest to determine whether long-term epigenetic modifications in offspring which are brought about during GDM may be a consequence of deregulated expression of HMG20A during pregnancy (see Section 3 and Figure 1).

We have also recently reported the expression of HMG20A murine astrocytes which is significantly increased in mice fed a high-fat diet (HFD) [115]. This increase in *Hmg20a* expression correlates with the stimulation of astrogliosis, a neuron-protective inflammatory process initiated in astrocytes [115,116]. Noteworthily, transcriptome profiling of primary astrocytes after *Hmg20a* silencing revealed a decrease in the inflammatory response (unpublished data). These data suggest that this chromatin remodeling factor is a common master regulator in β-cells, neurons and astrocytes with functional properties that integrate signals from altered glucose levels and stress situations favoring the establishment of adaptive responses to recover glucose homeostasis [116] (Figure 2).

### 5.3. Pax8, a Pregnancy-Dependent Islet Transcription Factor with Anti-Inflammatory Properties

PAX8 which is essential for the development and maintenance of the thyroid and the urogenital system was unexpectedly found as one of the most upregulated genes in mouse islets during gestation [117]. Surprisingly, *PAX8* expression has not been reported in the pancreas during development [89] and is not expressed in adult mouse islets while its transcript levels are barely detectable in adult human islets [118]. Furthermore, PAX8 was wrongfully detected in endocrine tumors of the pancreas raising doubts on the authenticity of *PAX8* expression in islets [118,119]. Nonetheless, we have recently validated that *Pax8* expression is robustly and transiently induced in mouse islets during gestation reaching maximal mRNA expression at gestational day 14.5, correlating with the end of the gestation-induced proliferation of β-cells [86,120]. Similarly, the treatment of human islets with prolactin, to mimic conditions of pregnancy during the 3rd trimester, induced a 2.5-fold increase in *PAX8* expression after 72h of treatment, correlating with the end of prolactin-induced β-cell proliferation [120]. These data support a role for PAX8 in the adaptation process ongoing during gestation to compensate for late pregnancy insulin resistance. Although the molecular mechanisms triggered by *PAX8* stimulation specifically during pregnancy are still unclear, its potential role in the adaptation process in response to insulin resistance is supported by a genome wide linkage and admixture mapping study that has found *PAX8* as a putative T2DM candidate gene in African Americans [121]. To elucidate the molecular pathways induced by PAX8 we performed whole transcriptome analysis of both human and mouse islets after lentiviral transduction with human or mouse PAX8 respectively. Despite differences between mouse and human transcriptome profiles, we detected an enrichment in immune-related diseases pathway, pinpointing at the role of PAX8 in immunity/inflammation in both species [120]. In agreement with this, a reduction in basal apoptosis, as well as protection from cytokine-induced apoptosis, was detected in islets transduced with PAX8. Due to the exclusive PAX8 expression during pregnancy and its potential implication in inflammation-related processed, we argued that detrimental SNPs within the *PAX8* gene may be associated with GDM. Consistent with this premise, we found 2 mutations within the *PAX8* gene, PAX8P25R and PAX8T356M in 2 independent families, in which females harboring one of these mutations developed GDM and glucose intolerance during pregnancies. Remarkably, one subject developed GDM in three consecutive pregnancies [120]. Functional analysis using reporter assays after lentiviral transduction of these *PAX8* variants in HEK293T cells demonstrated that both mutations hindered PAX8 transcriptional activity [120]. Based on the well-known function of PAX8 on thyroid gland development and maintenance it is not surprising that one of these GDM-associated mutations was also reported to cause congenital hypothyroidism. We presume that this association will also be established for the 2^nd^ mutation, PAX8T356M, however, since this is a novel mutation, nothing has yet been reported on its effect on the thyroid gland, nevertheless, the female harboring this mutation also exhibited gestational thyroid dysfunction [120]. Interestingly, and supporting the previously reported association of *PAX8* gene mutations with T2DM [121], the father of the female proband, also harboring the PAX8T356M mutation, developed T2DM and several familiar antecedents of T2DM were discovered in the family pedigree [120]. To study in detail the role of PAX8 we took advantage of the hemizygous *Pax8^+/−^* mice, since the KO model for this transcription factor results in neonatal death [122]. Unexpectedly, Pax8^+/−^ females remained normoglycemic and did not suffer glucose intolerance throughout pregnancy. Nevertheless, since TH levels in these animals remain unaltered when compared to control wild type (wt) littermates, we expect that a genetic compensation for Pax8 hemizygosity takes place in *Pax8^+/−^* animals, hindering the development of any alteration during pregnancy [120]. Nevertheless, male *Pax8^+/−^* mice (8 and 24 months-old animals) with age exhibit a mild but significant decrease in circulating T4 levels correlating with mild glucose intolerance and increased insulin resistance [123], a phenotype resembling T2DM. Additionally, these animals showed a reduction in the percentage of β-cells in the islets as an effect of the mild hypothyroidism that they develop with age since *Pax8* is not expressed in adult mice islets [123]. Moreover, in a comparative transcriptome analysis of the islets of *Pax8^+/-^* and wt mice we observed a decrease in master regulators of antioxidative processes, such as NRF2 in the hemizygous mice, supporting the requirement of adequate THs levels to preserve/protect β-cell integrity against oxidative stress damage [123]. Altogether these data suggest that PAX8 during pregnancy may have a dual role in glucose homeostasis: On one hand it will favor β-cell survival by enhancing β-cells resistance against apoptosis and stimulating the maintenance of a local anti-inflammatory milieu in the pancreas while on the other hand ensure systemic levels of TH, an important stimulator of insulin secretion and glucose clearance as well as protecting β-cells from environmental stressors (Figure 2).

### 5.4. The Interplay between PAX4, HMG20A and PAX8, in β-Cell Adaptation/Protection from GDM: Proposed Model of Action

**Figure 3:**
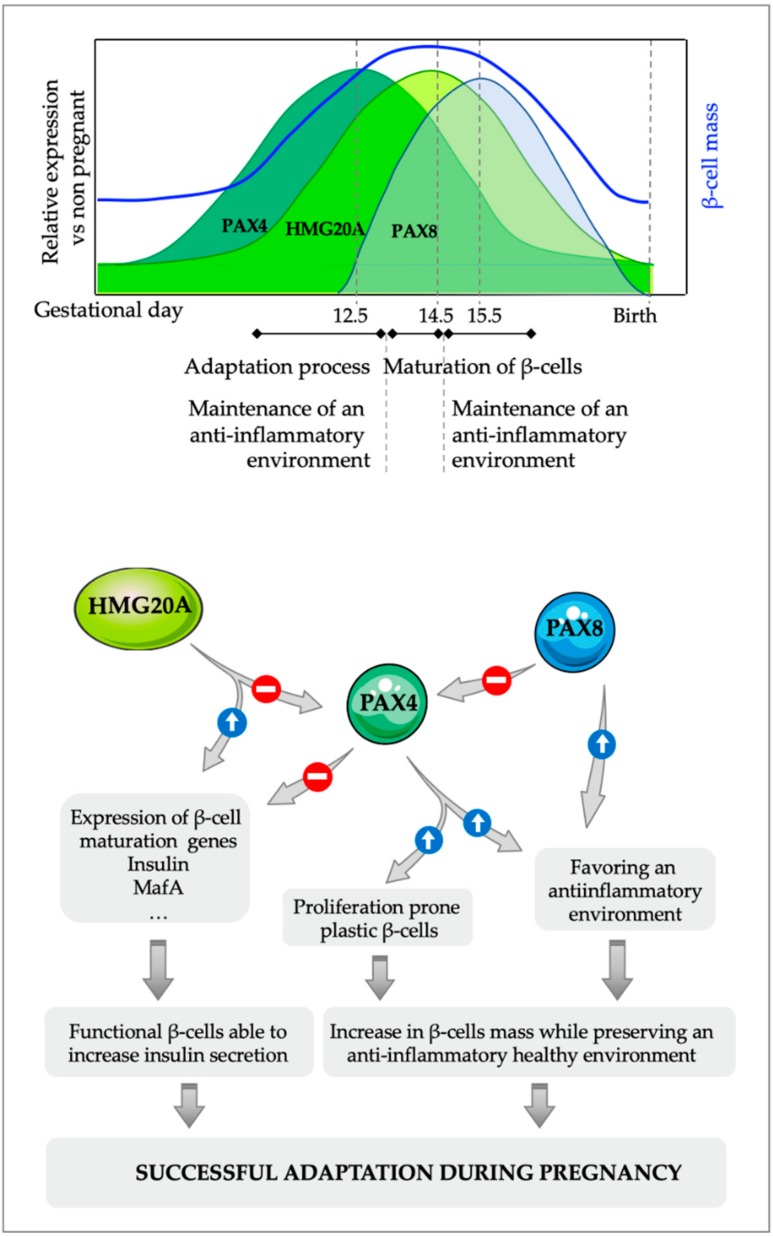
Proposed molecular model for the interaction between PAX4, HMG20A and PAX8 in pancreatic β-cells during islet adaptation in response to pregnancy and inflammation. The initial increase in PAX4 during pregnancy that allows the expansion of the β-cell mass is followed by a subsequent increase in the expression of the chromatin remodeler factor HMG20A, which downregulates *Pax4* expression. The decrease in PAX4 levels is necessary for the acquisition of a fully mature phenotype of these newly formed β-cells, which can then boost insulin expression/secretion to compensate for the increase in insulin demand that takes place during pregnancy. Additionally, PAX8, which is only expressed in islet during gestation, can blunt PAX4 activity through protein-protein interactions, further enhancing the inhibitory action of HMG20A on PAX4, allowing a faster maturation of the young β-cells. The combined anti-inflammatory actions of PAX4 and PAX8 will ensure a local permissive environment for the maintenance of a functional β-cell mass.

The specific islet regulation of the expression of these three factors, PAX4, HMG20A and PAX8 during pregnancy, together with the interactions among them have led us to propose a putative mechanistic model for the action of these factors during the islet adaptation process triggered by pregnancy (Figure 3).

The increased insulin demand that takes place during pregnancy, together with placental signals, triggers the expression of PAX4, which confers a more plastic phenotype to β-cells, allowing the initiation of the expansion of the β-cell mass, concomitantly with the increased resistance of these cells against environmental stressors. Although PAX4 *per se* might not be sufficient to increase the entire β-cell population [87], it allows the expansion of a proliferation prone subpopulation of β-cells [86]. Nevertheless, the acquisition of a fully mature phenotype of these new β-cells requires the decrease in PAX4 to allow expression of genes such as *Ins* and *MafA* required for the functional maturation of β-cells. This is achieved, at least in part, through the increase in HMG20A that in one hand downregulates the expression of *Pax4* while on the other hand enhances the expression of β-cell maturity genes such as *Ins* and *MafA* [108]. Additionally, the expression of PAX8 just after the increase in HMG20A levels will likely repress PAX4 transcriptional activity. Indeed, the high homology between the Paired domain (PD) of PAX8 with the PD of PAX6 (more than 70% homology at amino acid sequence) suggest that PAX8, similar to PAX6 [124] can interact with PAX4 through its PD and impair PAX4 transactivation, further inhibiting PAX4 activity and releasing the repression of β-cell maturity genes. The combined inhibitory action of HMG20A and PAX8 on PAX4 expression/activity will lead to the maturation of β-cells functionally able to address increased demands for insulin that takes place during the relatively short period of pregnancy. Additionally, the combined anti-inflammatory actions of both PAX4 and PAX8 ensure the maintenance of a permissive healthy environment during the entire pregnancy that protects β-cells from environmental stressors, allowing the maintenance of their functionality. In agreement with this model, alterations in the expression/functionality in any of these three factors will render an incomplete adaptation of the β-cells during pregnancy triggering the development of GDM.

## 6. Conclusions

The transitory increase in insulin resistance during pregnancy activates adaptation processes in β-cells to enhance insulin secretion and maintain normoglycemia. SNPs, mutations and/or epigenetic alterations in β-cell genes will impair adequate adaptation resulting in hyperglycemia and GDM development with the subsequent detrimental effects to both the mother and the offspring. This failure in the adaptation process is worsened by additional factors such as obesity, older age of pregnancy and inflammation processes. Therefore, under these conditions, the presence of mutations in important genes for β-cell formation/functionality such as *PAX4* (MODY9), *HMG20A* and *PAX8*, that may remain undiagnosed since their effects will not be revealed in the absence of environmental stressors, could trigger an inadequate response under situations of increased insulin demand such as pregnancy, triggering the development of GDM. Moreover, these mutations will also render the individuals more susceptible to the development of T2DM later on in life. As such, we would like to recommend that genetic screenings in women with GDM should include *PAX4*, *HMG20A* and *PAX8* to resolutely establish the correlation of genetic variants with this form of diabetes and potentially develop personalized therapy targeting specific pathways implicated in islet mass expansion/survival including potentiating the inflammatory response.

## Abbreviations

GDM	Gestational Diabetes Mellitus
T2DM	Type 2 Diabetes Mellitus
MODY	Maturity Onset Diabetes of the Young
MIA	Maternal Immune Activation
DOHaD	Developmental Origins of Health and Disease
TH	Thyroid Hormone
GWAS	Genome Wide Association Studies
SNP	Single Nucleotide Polymorphism
IL	Interleukin
IL-1Ra	Interleukin 1 Receptor antagonist
STZ	Streptozotocin
LSD1	Lysine-Specific Demethylase 1 A
